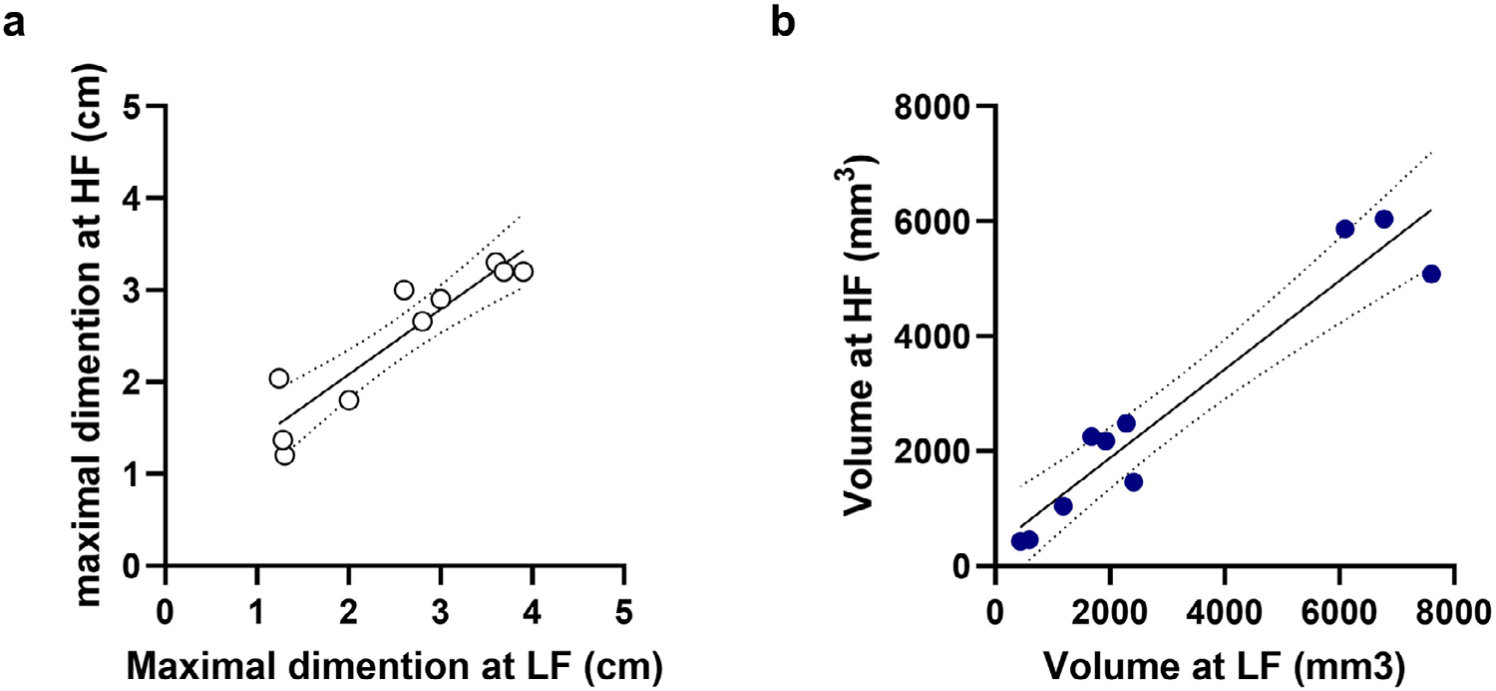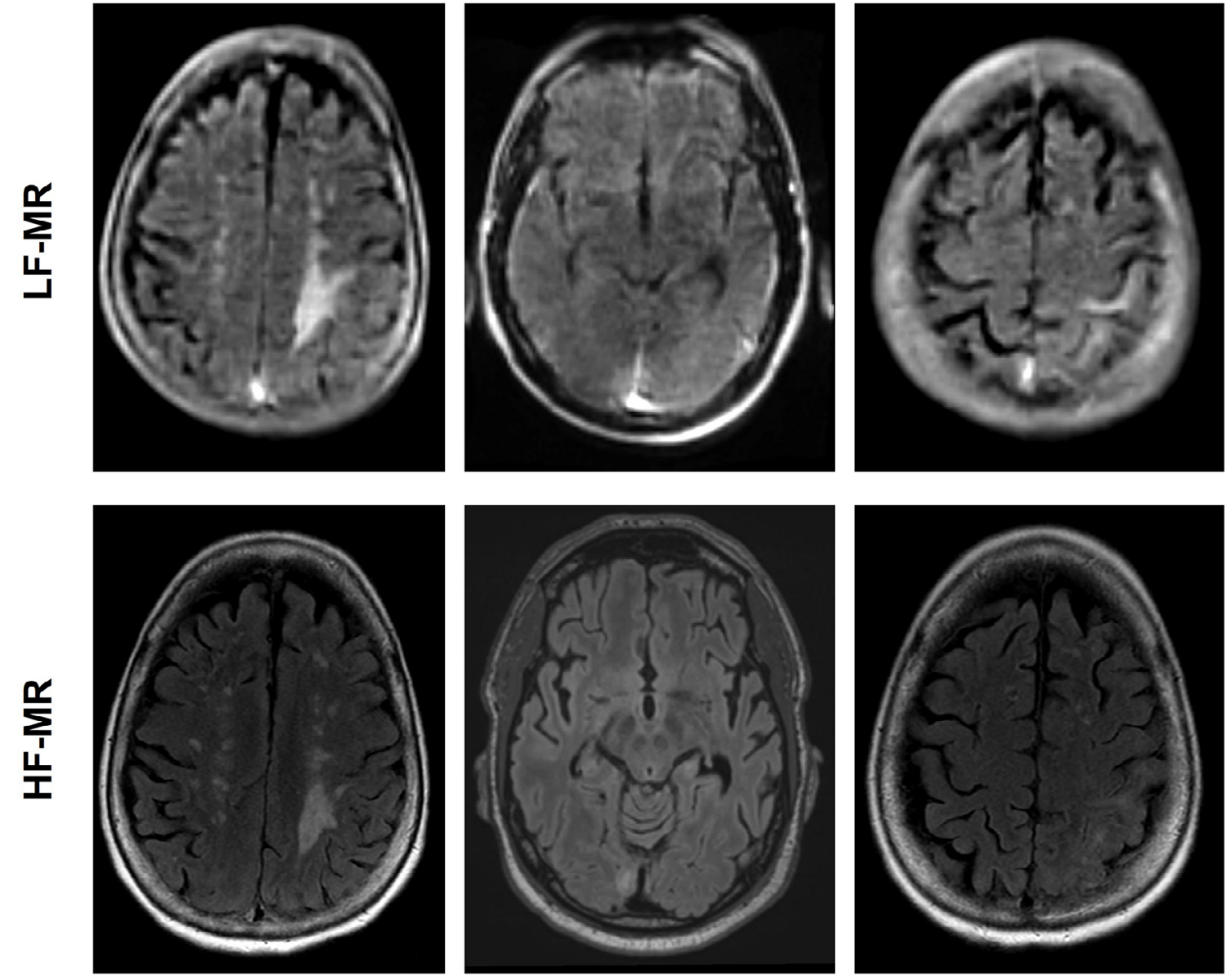# Portable Low‐Field MRI for Rapid Detection and Monitoring of ARIA in Lecanemab‐Treated Alzheimer's Disease Patients: A Pilot Study

**DOI:** 10.1002/alz70857_100090

**Published:** 2025-12-24

**Authors:** Shenghua Zhu, Jeremy N. Ford, Jarrel Seah, Saurabh Rohatgi, Esteban Calle Cadavid, Javier Romero

**Affiliations:** ^1^ Massachusetts General Hospital, Boston, MA, USA

## Abstract

**Background:**

Amyloid‐related imaging abnormalities (ARIA), particularly ARIA‐E (edema), are known complications in patients receiving lecanemab, an anti‐amyloid monoclonal antibody for early Alzheimer's disease (AD). Rapid detection and monitoring of ARIA are vital for patient safety and treatment optimization. However, timely access to conventional high‐field (HF) MRI is often limited by logistical and cost constraints.

**Method:**

From November 2023 to November 2024, 20 patients with early AD receiving lecanemab underwent a total of 40 low‐field (LF) MRI scans at 0.064 T (Swoop, Hyperfine). The high‐field MRI (3 T) study closest in time to each LF MRI served as the reference standard. Four board‐certified neuroradiologists, blinded to the HF MRI results, independently evaluated the LF MRI scans for ARIA‐E. ARIA‐E lesion dimensions and volumes were manually segmented on both LF MRI and HF MRI for correlation analysis.

**Result:**

Raters correctly identified ARIA‐E lesions in 2 of 20 patients, and accurately identified ARIA‐negative cases in 18 of 20 patients. Manually segmented ARIA‐E maximal dimension on LF MRI strongly correlated with HF MRI measurements (*r* = 0.9239, *p* =  0.0001), and volumetric analyses likewise showed a high correlation (*r* = 0.9562, *p* < 0.0001).

**Conclusion:**

This pilot study suggests that portable LF MRI can detect and monitor ARIA‐E in patients treated with lecanemab, potentially circumventing delays associated with HF MRI. Although image quality is lower than HF MRI, the ability to guide therapeutic decisions promptly may prove clinically significant. Further larger‐scale studies are warranted to validate these initial findings, refine imaging protocols, and explore the broader role of LF MRI in routine ARIA screening and follow‐up.